# Comparison of Postoperative Complications and Reoperation Rates of Le Fort I Osteotomies Using Demineralized Bone Matrix (DBM) or Autogenous Bone Grafts in Patients with Orofacial Clefts and Craniofacial Malformations

**DOI:** 10.3390/dj13060256

**Published:** 2025-06-09

**Authors:** Noémi Sipos, Junnu Leikola, Arja Heliövaara, Eeva Kormi, Juho Suojanen

**Affiliations:** 1 Cleft Palate and Craniofacial Centre, Department of Plastic Surgery, Helsinki University Hospital and University of Helsinki, 00029 Helsinki, Finland; noemi.sipos@outlook.com (N.S.);; 2 Päijät-Häme Joint Authority for Health and Wellbeing, Department of Oral and Maxillofacial Surgery, Päijät-Häme Central Hospital, 15850 Lahti, Finland; 3 Clinicum, Faculty of Medicine, University of Helsinki, 00014 Helsinki, Finland

**Keywords:** DBM (demineralized bone matrix), DBX (demineralized bone matrix), autogenous bone graft, osteotomy, Le Fort I, complications, reoperations, cleft, craniofacial, malformation

## Abstract

**Background:** This study aims to evaluate surgical outcomes and compares the prevalence and severity of postoperative complications and reoperations with maxillary osteotomies, focusing on the effectiveness of fixation with demineralized bone matrix (DBM) versus autogenous bone grafts (ABG) in patients with orofacial clefts and craniofacial malformations. **Methods:** This retrospective cohort study included 138 consecutive patients treated at the Cleft Palate and Craniofacial Center, Helsinki University Hospital, from 2014 to 2022. The cohort consisted of patients with clefts (*n* = 113), craniosynostosis, and craniofacial syndromes (*n* = 25). The DBM group (*n* = 103) received DBX^®^ (Musculoskeletal Transplant Foundation, Edison, NJ, USA), while the ABG group (*n* = 35) received autogenous bone grafts. Surgical procedures included Le Fort I and bimaxillary osteotomies. Complications involving the maxilla or both jaws were included in the analysis. Both major and minor complications, as well as reoperations, were analyzed and compared. **Results:** The DBM group had 13.6% of patients with complications, while the ABG group had 20.0%. Reoperation rates were 6.8% for the DBM group and 5.7% for the ABG group. There were no statistically significant differences in complication or reoperation rates between the DBM and ABG groups. **Conclusions:** The findings suggest that using DBM or ABG in maxillary osteotomies does not significantly affect complication or reoperation rates, supporting DBM as a viable alternative for maxillary surgeries.

## 1. Introduction

Patients with cleft lip and/or palate (CL/P) and craniofacial malformations often require maxillary advancement surgery to address maxillary hypoplasia, improve facial esthetics, and correct functional issues such as crossbite. Orthognathic surgery (OGS) is beneficial in this patient group, as it enables correction of skeletal deformities including maxillary retrusion, vertical and horizontal discrepancies, and malocclusion [1]. CL/P patients typically require multiple surgeries to achieve desired functional and esthetic outcomes, and the severity of the cleft type correlates with the likelihood of requiring OGS [2]. Studies show that OGS provides anatomical benefits and enhances self-esteem [3]. The need for surgery varies by cleft type: 40.0–48.3% of unilateral cleft lip and palate (UCLP) patients and up to 65.1–66% of bilateral cleft lip and palate (BCLP) patients undergo OGS [4,5,6]. Among isolated cleft palate (CP) patients, surgical needs range from 4 to 13.2% [7,8,9]. Although smaller studies report varying results, their limited sample sizes reduce their reliability, and their findings should be interpreted with caution.

Common complications associated with Le Fort I osteotomies in patients with clefts, craniofacial malformations, or syndromes include intraoperative hemorrhage, postoperative surgical relapse, secondary malocclusion, fistula formation, loss of dental vitality, and necrosis of the maxillary segment [10,11]. These complication rates are influenced by anatomical malformations and functional impairments inherent in cleft patients [7,12]. CLP often presents with maxillary hypoplasia, which can result in class III malocclusion due to excessive mandibular rotation and a reduction in facial height [13]. The most frequent intraoperative issue encountered in patients with CLP is hemorrhage [12]. 

Reoperations primarily occur due to iatrogenic malposition, while approximately 30% of cases involve relapse to the presurgical deformity [14]. Additional reasons for reoperations after Le Fort I surgery, aside from skeletal relapse, include malocclusion, asymmetry, maxillary deformities, and hardware plate fractures [15].

Graft materials for Le Fort I surgeries include autogenous, allograft, and alloplastic options. Autogenous bone grafts can be harvested from the mandible, rib, iliac crest, and calvarial bones, the iliac crest being the most common harvesting place [16,17]. Autogenous bone grafts, though osteoinductive and osteoconductive, require additional surgical sites, increasing donor site morbidity [16,18,19]. Other potential complications of autogenous bone graft include neurovascular damage, avulsion fractures, hematoma, and damage to the sacroiliac joint and ureter [20,21]. Considering the surgical costs associated with an additional donor site and prolonged anesthesia time, DBM emerges as a more cost-effective option. Additionally, patient recovery tends to be notably easier due to the less invasive nature of the procedure.

Demineralized bone matrix (DBM), a group of allograft substitutes, includes products like DBX^®^ (Musculoskeletal Transplant Foundation, Edison, NJ, USA), Grafton^®^ (Osteo Tech, Eatontown, NJ USA), and Allomatrix^®^ (Wright Medical Technology Inc., Memphis, TN, USA). DBM is created from freeze-dried bone taken from cadavers, demineralized, and suspended in a synthetic biological carrier, resulting in a biocompatible material [17]. It is available in various forms such as putty, gel, and chips [17]. Allografts such as DBM offer osteoinductive and osteoconductive properties and are readily available, though their effectiveness in maxillary surgeries is less explored [17,22]. DBM complications in maxillofacial surgeries include sinusitis, delayed ossification, and necrosis of an upper canine, as well as infection and occlusal malalignment [17,23].

Alternative graft materials include alloplastic grafts such as ProPlast blocks, solid block hydroxyapatite, and porous block hydroxyapatite (PHBHA) [17]. Additionally, xenograft materials like bovine (Bio-Oss^®^; Geistlich Pharma AG, Wolhusen, Switzerland) or porcine bone scaffolds are commonly used [23]. While bovine bone materials are widely used in oral and maxillofacial surgery, their effectiveness in Le Fort I osteotomy gaps remain less explored. A study conducted on 25 patients undergoing Le Fort I surgery concluded that there were comparable outcomes in mineralized fraction and postoperative stability between autogenous iliac crest grafts and Bio-Oss^®^ blocks [24]. Alloplastic and xenogenic grafts are readily available but lack osteoinductive properties, resulting in slower healing and may pose infection risks and healing complications due to their slow resorption [17].

The primary aim of this study is to compare the prevalence and severity of peri- and postoperative complications, including reoperations, between patients receiving DBM and those receiving ABG in maxillary osteotomies. Our findings aim to guide graft selection and enhance surgical outcomes in patients with clefts and craniofacial malformations or syndromes.

## 2. Materials and Methods

This retrospective cohort study included 138 consecutive patients treated at the Cleft Palate and Craniofacial Center, Helsinki University Hospital, Finland (Cleft Palate and Craniofacial Center, Department of Plastic Surgery) from 2014 to 2022. All surgeries were performed by five senior cleft surgeons. Clinical findings and adverse effects related to osteotomy surgery were recorded in electronic medical records by both the operating surgeon and orthodontist following the initial procedure and during subsequent follow-ups.

### 2.1. Surgical Details 

All patients underwent a Le Fort I or bimaxillary osteotomy under general anesthesia. Routine presurgical planning involved a surgeon and an orthodontist. During surgery, patients received personalized 3D-planned plates or conventional mini plates. Intravenous antibiotics were routinely used perioperatively to reduce infection risks. Patients received a standard dosage of antibiotics. DBM used in this study was DBX^®^. The ABG used in our study consisted of a cortico-cancellous bone block harvested from the medial aspect of the iliac crest. The graft was then secured to the osteotomy line using mini screws. All operations were performed employing the same technique and the same type of ABG. Alloplastic materials were not used in our hospital during our study period.

### 2.2. Ethical Approval

This retrospective study was conducted in accordance with the Declaration of Helsinki and approved by the Institutional Committee of Helsinki University Hospital (HUS/917/2021).

### 2.3. Data Collection

All patient data were retrieved from electronic medical records from 2014 to 2022 and retrospectively analyzed. Exclusion criteria included lack of bone graft, incomplete surgical details or medical records, and surgeries outside the 2014–2022 timeframe. Demographic data collected included gender and age at the time of surgery. Preoperative information included the diagnosis leading to surgery, the type of bone graft used, complications occurring during surgery or follow-ups, and the occurrence of reoperations (Table 1). Postoperative data described the rate and nature of detected complications. Patients who experienced peri- or postoperative complications were further investigated. Complications were categorized into immediate major and minor, as well as delayed major and minor, and further classified according to the Clavien-Dindo system [25,26]. Certain postoperative findings were considered expected side effects of OGS and not classified as complications. These included, for example, prolonged numbness in the surgical area, clicking of the temporomandibular joint during mouth closure, minor edge necrosis of the wound, dehiscence, mucous membrane disruption in the nasal area during osteotomy, mucosal discharge, and immediate postoperative nausea and vomiting. Velopharyngeal impairment was also not classified as a complication in this study, as it is well established that Le Fort I osteotomy can influence velopharyngeal function in patients with clefts. Patients with preoperative borderline or more severe velopharyngeal incompetence are particularly at risk [27].

### 2.4. Study Population

A total of 138 consecutive patients were included in this study, with 103 receiving (DBM) and 35 receiving ABG. The study population comprised 72 females and 66 males, with a median age of 20.4 years (Table 1). Le Fort I was the primary surgical approach in 84.8% of cases, while bimaxillary osteotomy was performed in 16.0% of cases.

The DBM group included considerably more patients than the ABG group, with 103 patients compared to 35. DBM gradually replaced ABG after 2016 due to favorable outcomes and practice changes, leading to a larger DBM sample in this complete cohort. Despite this difference in numbers, the demographics of both groups were quite similar. The median age was comparable in both groups, and the gender distribution was approximately 50% male and 50% female. The DBM group had a slightly higher number of patients with clefts, which was the predominant category in both groups. All demographic details are presented in Table 1. The cleft category included patients with cleft palate, cleft lip, or both cleft lip and palate. The craniofacial malformations category comprised patients with craniofacial malformations, craniosynostoses, and syndromes like Goldenhar syndrome.

### 2.5. Statistical Analysis

Statistical analysis was made with SPSS v28 (IBM). The continuous variables were tested with Shapiro–Wilk test for normality. The difference between the DBM and the ABG group was analyzed with the Chi-Square test and logistic regression. Further analysis of continuous variables utilized non-parametric Mann–Whitney U-test as the data did not fit to a normal distribution. The significance was set to *p*-value < 0.05.

For statistical analysis, diagnoses were divided into two categories: “cleft” and “other,” to ensure that each group had a sufficient size for evaluation. Complications were classified according to the Clavien-Dindo system. If a patient experienced both a minor and a major complication, the case was categorized based on the highest Clavien-Dindo grade.

## 3. Results

The DBM and ABG groups showed no statistical difference in age (*p* = 0.939), sex (*p* = 0.772), or diagnosis (0.063) (Table 1).

The complication rate was similar in both study groups (*p* = 0.416) (Table 2). Sex (*p* = 1), diagnosis (*p* = 0.538), or age (*p* = 0.965) did not predict the complications either (Table 3). Also, severity of the complications shows no statistical difference across the study groups (*p* = 0.400) (Table 4).

### 3.1. Postoperative Complications

The DBM group had 13.6% of patients with complications, while the ABG group had 20.0%. Some patients experienced multiple complications, leading to an overall complication rate of 18.8% for Le Fort I and bimaxillary surgeries, with rates of 17.5% in the DBM group and 22.9% in the ABG group. Immediate major complications occurred in 1.4% of cases, and minor complications in 7.3%. Delayed major complications were seen in 3.6% and delayed minor complications in 6.5%. (Table 2).

Both groups experienced one immediate major complication (1.0% for DBM, 2.9% for ABG). Immediate minor complications occurred in 7.8% (eight cases) of the DBM group and 5.7% (two cases) of the ABG group. Delayed major complications were observed in 3.9% (four cases) in the DBM group and 5.7% (two cases) in the ABG group. Delayed minor complications occurred in 4.9% (five cases) of the DBM group and 8.6% (three cases) of the ABG group (Table 2).

Most complications in our study were classified as Clavien-Dindo stages I or II. However, two cases (one from each group) were categorized as stage IV due to major postoperative bleeding—representing 1.0% in the DBM group and 2.9% in the ABG group. Other notable findings included two absent seizures in the DBM group. In addition, there were five fistula cases in the DBM group, with two requiring additional surgeries.

Our findings reveal that the ABG group had a higher overall complication rate compared to the DBM group. ABG patients experienced more complications in all subcategories except for immediate minor complications, where the DBM group had a slightly higher rate. However, statistical analysis indicated no significant differences in complication rates between the groups.

### 3.2. Reoperations

The overall reoperation rate due to complications following initial Le Fort I or bimaxillary surgery was 6.5% (9 out of 138 patients). In the DBM group, complications led to reoperations in 7 out of 103 patients, resulting in a reoperation rate of 6.8%. In contrast, the ABG group had 2 reoperations out of 35 patients, yielding a reoperation rate of 5.7%. The reasons for reoperation included the removal of osteosynthesis material, re-osteosynthesis, fistula closure, corrections of malocclusion, and fat transfer due to exposed fixation plates (Table 5). The reoperation rate was slightly higher in the DBM group (6.8%) compared to the ABG group (5.7%).

When analyzing the number of patients who experienced at least one complication, the DBM group had a lower rate (13.6%) compared to the ABG group (20.0%). The overall complication rate was also higher in the ABG group (22.9%) than in the DBM group (17.5%). Despite these differences, statistical analysis revealed no significant difference between the two groups.

The rates of reoperation were comparable, with the DBM group experiencing a rate of 6.8% and the ABG group at 5.7%. Additionally, none of the variables tested could account for the occurrence of complications, and according to our finings, the severity of these complications was not influenced by the choice of osteotomy material.

## 4. Discussion

Our findings reveal no statistically significant differences in postoperative complications or reoperation rates between ABG and DBM in maxillary osteotomies. The statistical analysis focused solely on distinguishing between minor and major complications, without accounting for whether complications occurred immediately or were delayed. Notably, a meta-analysis by Valls-Ontanon (2021) supports our results, demonstrating that bone grafting does not substantially influence the overall complication rate in patients with cleft lip and palate (CLP) [28]. Additionally, our analysis indicates that age is not a significant predictor of complication rates.

ABG is widely regarded as the gold-standard bone graft material for cleft patients, but there is limited research available on the risk profiles, complications, and outcomes of OGS using DBM. DBM is more readily available than ABG and reduces surgical morbidity, as it does not require an additional surgical site. Although the material costs of DBM may be higher, a study by Mehta et al. (2018) demonstrated its greater overall cost-effectiveness compared to iliac crest bone grafting [29]. This was attributed to significantly shorter anesthesia times, reduced pharmacy and operating room expenses, and a notably shorter operative duration—reduced by approximately 30 min [29]. Inpatient stays were also reduced to nearly one-third. Overall, the total treatment cost for alveolar cleft deformities was around 30% lower in the DBM group than in the ABG group [29].

### 4.1. Postoperative Complications

Our study found an overall complication rate of 18.8%. This rate is consistent with the 12.8% rate, reported in Yamaguchi et al.’s 2016 systematic review, but it contrasts sharply with the much lower rate of 4.2% identified in Cheung and Chua’s 2006 meta-analysis on cleft patients [30,31].

Patients with CLP often have considerable scarring from previous soft tissue surgeries and require substantial maxillary advancement, increasing surgical complexity and risk [32]. Patients with BCLP commonly face challenges such as missing lateral incisors, alveolar irregularities, nasal asymmetry, and weakened upper lip muscles. Furthermore, intrinsic embryologic factors, along with scarring from previous surgeries, can restrict maxillary growth and overall development [12]. Despite these challenges, the incidence of major complications remained low in our study. Notably, this increased complexity does not inherently lead to higher complication rates compared to orthognathic procedures in non-cleft patients [30]. However, the variability in complication rates observed in previous studies underscores the need for further investigation in this area.

According to the literature, hemorrhage is the most commonly observed complication following Le Fort I osteotomy [33]. Despite instances of major bleeding, the occurrence of life-threatening hemorrhage remains low, with reported rates of 0–0.7% [33]. In the study, both groups experienced one case of major bleeding, which accounted for 1.0% in the DBM group and 2.9% in the ABG group. Kramer et al. (2004) reported a higher incidence of major bleeding (5.2%) in patients with considerable anatomical variations [10]. Major bleeding is often associated with damage to the descending palatine artery during the separation of the pterygomaxillary junction [33]. In the DBM group, bleeding occurred postoperatively when a bone fragment was dislodged through the osteotomy line after a violent sneeze. In the ABG group, unexplained intraoperative bleeding resulted in delayed extubation.

Two cases of absent seizures were observed in the DBM group, one of which was potentially linked to opioid use.

Incomplete ossification of the osteotomy gap is a considerable concern in osteotomies like Le Fort I [17,18]. Cleft patients frequently undergo multiple facial surgeries, leading to fibrosis and scarring that complicate maxillary movement during Le Fort I osteotomy and increase the risk of skeletal relapse [2]. Preoperatively, cleft patients typically display considerable maxillary hypoplasia, necessitating larger advancements during surgery, thereby increasing relapse rates compared to non-cleft patients [34]. Two systematic reviews have identified maxillary relapse as the most common complication among cleft patients, with relapse rates exceeding 50% [31,32]. Yamaguchi et al. (2016) reported a relapse rate of 53.3%, while Zaroni et al. (2024) observed a similar rate of 51.3% [30,32]. In our study, we observed three case of maxillary relapse among the 26 complications, with an incidence of 11.5%. The relapses were classified as malocclusions in our study. One relapse occurred in the DBM group and two in the ABG group. The relapse in the DBM group does not appear to be directly related to the use of DBM, but it may have been influenced by the use of thin plates during surgery, which could have contributed to the complication. The DBM patient also had BCLP, a condition strongly associated with an increased risk of skeletal relapse due to the extensive surgical movements required [35]. Hirano and Suzuki’s (2001) study found a significant correlation between BCLP and relapse complications in OGS, further supporting the increased risk of relapse in patients with BCLP [35]. This suggests that the patient’s cleft type and choice of fixation plates, rather than the use of DBM, were likely the main factors contributing to the relapse. One patient in the ABG group diagnosed with Goldenhar syndrome likely experienced an open bite due to temporomandibular joint (TMJ) incompetence, accentuated by a retrognathic mandible. The etiology of malocclusion, in this case, may be attributed partly to surgical intervention and partly to inherent jaw disproportion. Both malocclusions in the ABG group were corrected by bilateral TMJ prostheses.

The choice of fixation plates (3D versus mini plates) may have contributed to other complications observed in our study, such as plate exposure and surface tenderness, which were found exclusively in the DBM group. However, the correlation between plate type and complication rates was not further analyzed in this study.

A complication unique to the ABG group is donor site pain from bone harvesting, which is absent in the DBM group. This pain occurred in 1 patient out of 35 patients (2.86%). This rate is relatively low compared to a study by Laurie et al. (1984), which reported wound complications in 21% of cases involving autogenous bone harvesting from the iliac crest area [16]. However, it is important to note that the higher incidence in Laurie et al.’s study was associated with direct iliac crest incisions, whereas no complications were reported in cases where the incisions were made above or below the crest [16]. In our study, all bone grafts in the ABG group were harvested from the iliac crest, though we did not analyze the donor sites in further detail. The patients in our study came from across the country, meaning that small postoperative wounds and delayed complications were managed at their local hospitals or healthcare centers. Permanent complications would have appeared in the electronic patient records.

DBM is a malleable substance without rigid fixation; therefore, it theoretically poses a potential risk for sinus infections. However, our research found no significant association between DBM usage and sinusitis, since no patient in our research group presented with sinusitis. Contrary to our supposition, a retrospective study conducted by Lye et al. (2008) found a higher incidence of sinusitis in the non-DBX^®^ group compared to the DBX^®^ group [17]. However, their study did not focus on cleft and craniofacial malformation patients, and complications in this group may vary from non-cleft patients.

### 4.2. Reoperations

The DBM group had a slightly higher reoperation rate than the ABG group. In the DBM group, additional reoperations were due to fistula closures and exposed fixation plates. Meanwhile, the ABG group experienced reoperations primarily due to malocclusion, necessitating further surgical correction. Four cases out of 103 (3.9%) were somewhat related to the osteotomy material. Among the two cases of non-ossification in the DBM group, one was likely influenced by the patient’s diabetes, a condition known to delay ossification. The second case involved a patient with a fractured fixation plate, which required reoperation due to non-ossification; however, no evidence of osteolysis was observed. Interestingly, no cases of non-ossification were observed in the ABG group. This discrepancy may be explained by differences in sample sizes between the groups and the comorbidities present in the patients. However, this was not specifically analyzed. In other studies, DBM has demonstrated similar effectiveness in promoting bone growth as ABG [17]. Francoisse et al. (2023) found comparable union rates between DBX^®^ and autogenous bone grafts in a retrospective study involving 17 cleft alveolus patients undergoing revision surgery for bone nonunion [36].

### 4.3. Strengths

This study included a larger number of patients compared to other research in this field. Moreover, all patients underwent surgery in the same hospital. Research on the use of DBM in OGS is limited, particularly regarding patients with cleft and craniofacial deformities. This patient group presents unique challenges that distinguish them from those without facial anomalies. By comparing DBM to ABG, we can gain valuable insights that may guide surgical decision-making and material selection, potentially leading to less invasive and more effective treatment options for these complex cases. This study serves as a solid foundation for future research on the long-term efficacy of (DBM).

### 4.4. Limitations

The limitations of this study include the heterogeneity of the study population. Additionally, the involvement of various surgeons and assistant surgeons may have influenced the outcomes. Nevertheless, the surgical methods employed were standardized and comparable, thereby minimizing the impact of differing operating surgeons on the results. No specific timeframe was established for identifying delayed complications. The patients from the last decile were operated on near the time of data collection; thus, some delayed complications may have been missed. Cephalometric data were unavailable, so the impact on skeletal stability was not analyzed in detail. Patients’ comorbidities were not discussed in detail in the statistical analysis, but they were factored into the discussion of complications and reoperations in certain cases.

## 5. Conclusions

Our study demonstrates no statistically significant difference in the risk of complications or reoperations between DBM and (ABG) in maxillary osteotomies. This finding aligns with previous research highlighting DBM’s osteoinductive and osteoconductive properties. Our results support the continued use of DBM as a viable alternative to ABG in craniofacial surgeries for patients with orofacial clefts and craniofacial malformations. Nonetheless, given the limited number of comparative studies in this area, further research is essential to elucidate long-term outcomes. The limitations of this study, along with the substantial difference in sample sizes, must also be considered when interpreting the conclusions.

## Figures and Tables

**Table 1:** Demographics.

	All (n)	All (%)	DBM (n)	DBM (%)	ABG (n)	ABG (%)	*p*-Value	Sig.
**n**	138		103		35			
**Age (years)**	20.4		20.5		20.4		0.939	NS
**Sex**							0.772	NS
Female	72	52.2	53	51.5	19	54.3		
Male	66	47.8	50	48.5	16	45.7		
**Diagnosis**							0.063	NS
Cleft	113	81.9	88	85.4	25	71.4		
Craniofacial malformation or syndrome	25	18.1	15	14.6	10	28.6		

DBM group (demineralized bone matrix), ABG group (autologous bone graft), and NS (non-significant).

**Table 2:** Postoperative complications.

Complication Categories	Clavien-Dindo	DBM Group	ABG Group	Total
**Immediate major**		**1 (1.0%)**	**1 (2.9%)**	**2 (1.4%)**
Post-operative bleeding	IV	1	1	
**Immediate minor**		**8 (7.8%)**	**2 (5.7%)**	**10 (7.3%)**
Fistula	I, IIIb	5	0	
Absence seizure	II	2	0	
Donor site pain	I	0	1	
Transient dysphagia	I	0	1	
Wisdom tooth raised near the sinus	I	1	0	
**Delayed major**		**4 (3.9%)**	**2 (5.7%)**	**6 (4.3%)**
Non-ossification	IIIb	3	0	
Malocclusion	I, IIIb	1	2	
**Delayed minor**		**5 (4.9%)**	**3 (8.6%)**	**8 (5.9%)**
Exposed fixation plate	IIIb	3	0	
Plate surface tenderness	IIIb	1	0	
Bone marrow defect	IIIb	1	0	
Nasal congestion	I	0	1	
Root canal injury	I	0	2	

**Table 3:** Subgroup analysis of complications was performed to evaluate the impact of different variables on complication rates.

	Complications (n)	Complications (%)		*p* Value	Sig.
**Intervention**				0.416	NS
DBM	14	13.6			
ABG	7	20.0			
**Sex**				1	NS
Female	11	15.3			
Male	10	15.2			
**Diagnosis**				0.538	NS
Cleft	16	14.2			
Craniofacial malformation or syndrome	5	20.0			
	**Standard error**	**Wald**	**dF**		
**Age**	0.034	0.002	1	0.965	NS

DBM group (demineralized bone matrix), ABG group (autologous bone graft), and NS (non-significant).

**Table 4:** Complication severity (Clavien-Dindo) across the study groups.

Clavien-Dindo	DBM (n)	ABG (n)		
I	3	4		
II	2	0		
IIIa	0	0		
IIIb	8	2		
IV	1	1		
V	0	0		
			***p* value**	**Sig.**
**Significance**			0.400	NS

DBM group (demineralized bone matrix), ABG group (autologous bone graft), and NS (non-significant).

**Table 5:** Reoperations.

Reoperation-Related Complications	DBM Group	ABG Group	Total
	7 (6.8%)	2 (5.7%)	9 (6.5%)
Osteosynthesis material removal	1	0	
Re-osteosynthesis	3	0	
Fistula closure	2	0	
Correction of malocclusion	0	2	
Fat transfer due to exposed fixation plate	1	0	

## Data Availability

The data are not publicly available due to ethical and data protection regulations at the Helsinki University Hospital (HUS).
